# Stem/Progenitor Cells and Related Therapy in Bronchopulmonary Dysplasia

**DOI:** 10.3390/ijms241311229

**Published:** 2023-07-07

**Authors:** Manuela Marega, Natalia El-Merhie, Mira Y. Gökyildirim, Valerie Orth, Saverio Bellusci, Cho-Ming Chao

**Affiliations:** 1 German Center for Lung Research (DZL), Department of Pulmonary and Critical Care Medicine and Infectious Diseases, Cardio-Pulmonary Institute (CPI), Universities of Giessen and Marburg Lung Center (UGMLC), Justus Liebig University Giessen, 35392 Giessen, Germany; 2 Department of Pediatrics, Centre for Clinical and Translational Research (CCTR), Helios University Hospital Wuppertal, Witten/Herdecke University, 42283 Wuppertal, Germany; valerie.orth@helios-gesundheit.de; 3 Institute for Lung Health (ILH), Member of the German Center for Lung Research (DZL), Justus Liebig University Giessen, 35392 Giessen, Germany; 4 Department of Pediatrics, University Medical Center Rostock, University of Rostock, 18057 Rostock, Germany

**Keywords:** bronchopulmonary dysplasia, premature infants, lung injury, chronic lung disease, inflammation, stem cells, extracellular vesicles, tissue repair

## Abstract

Bronchopulmonary dysplasia (BPD) is a chronic lung disease commonly seen in preterm infants, and is triggered by infection, mechanical ventilation, and oxygen toxicity. Among other problems, lifelong limitations in lung function and impaired psychomotor development may result. Despite major advances in understanding the disease pathologies, successful interventions are still limited to only a few drug therapies with a restricted therapeutic benefit, and which sometimes have significant side effects. As a more promising therapeutic option, mesenchymal stem cells (MSCs) have been in focus for several years due to their anti-inflammatory effects and their secretion of growth and development promoting factors. Preclinical studies provide evidence in that MSCs have the potential to contribute to the repair of lung injuries. This review provides an overview of MSCs, and other stem/progenitor cells present in the lung, their identifying characteristics, and their differentiation potential, including cytokine/growth factor involvement. Furthermore, animal studies and clinical trials using stem cells or their secretome are reviewed. To bring MSC-based therapeutic options further to clinical use, standardized protocols are needed, and upcoming side effects must be critically evaluated. To fill these gaps of knowledge, the MSCs’ behavior and the effects of their secretome have to be examined in more (pre-) clinical studies, from which only few have been designed to date.

## 1. Introduction

Preterm birth is the leading cause of infant mortality and long-term morbidity and represents a significant healthcare burden [1,2,3]. Premature newborns may suffer from respiratory distress requiring mechanical ventilation and oxygen support. The prolonged use of oxygen increases its concentration in the lungs (hyperoxia), which can eventually lead to inflammation resulting in abnormal lung development [4,5]. The affected infants are at high risk to develop bronchopulmonary dysplasia (BPD), a multifactorial chronic lung disease characterized by immature lung vasculature, inflammation, restricted lung development, and disordered lung repair [6]. The lower the gestational age and birth weight, the higher the risk to suffer from clinical syndromes which are considered as BPD. To date, the pathologic description of BPD includes disrupted alveolarization and microvasculature development, fibrosis, and cystic emphysema, and is likely to emerge due to disturbed signaling pathways [7].

Alongside a number of postnatal challenges that contribute to the development of BPD, the results of several studies have implicated that adverse conditions during pregnancy like placental vascular disease, intrauterine growth restriction, or chorioamnionitis may already be sufficient to induce or at least promote BPD and its related consequences [8].

Several advances in perinatal and neonatal medicine have improved the survival rates of preterm babies. However, these infants are susceptible to short- or long-term respiratory complications and are at an increased risk of BPD [6]. The current therapy is limited to a few drugs, such as caffeine and vitamin A, or corticosteroids like dexamethasone. However, this pharmacological approach has been controversially discussed, since on the one hand it has been shown to be associated with neurodevelopmental complications [9,10], whereas on the other hand, a low-dose treatment of high-risk infants turned out to have a positive benefit vs danger relation [11,12]. Nevertheless, these drugs failed to address all aspects of the disease, including the impaired alveolarization and the inflammation. Thus, no successful handling is available to prevent the long-lasting consequences of BPD, like pulmonary hypertension, exercise intolerance with sometimes the need for life long oxygen supply, and echocardiographic abnormalities [13]. In this scenario, the need of alternative safe therapies has strongly emerged.

In BPD, inflammation is considered as an initiative characteristic, and infections are deemed as high-risk factors. The levels of proinflammatory cytokines and chemokines, like interleukin (IL)-1b, IL-6, IL-8, tumor necrosis factor (TNF)-α, MCP-1, -2, -3, and transforming growth factor (TGF)-β1 are all increased, while anti-inflammatory molecules, like IL-4, IL-13, and IL-10, growth factors (fibroblast growth factor 10 (FGF-10), and vascular endothelial growth factor A (VEGFA), and platelet-derived growth factor subunit A (PDGF-A) are all decreased, whereas they are required for the physiological development of the lung and its repair during injury [14]. Regarding inflammation, the nuclear factor-kappa B (NF-κB) pathway has been implicated in the pathogenesis of BPD. NF-κB is a transcription factor that plays a critical role in the regulation of the inflammation and cellular stress responses. In BPD, activation of the NF-κB pathway has been observed and linked to the persistent inflammation and oxidative stress that are characteristics of this disease [14].

Stem-cell based therapy has been proven to be promising. Stem/progenitor cells are important in preserving the structure of the lung, and their presence is fundamental for the repair of the damaged tissue in lung diseases.

Therapeutic approaches should therefore address the restoration of the function of the different stem/progenitor cells (as well as their presence) in their compartments, including growth factors modulating the inflammation derived from the damage. Interestingly, mesenchymal stem cells (MSCs) have recently emerged as a good candidate, as they support cells during their development [15,16]. Additionally, MSCs can modulate the immune response with a reduction in inflammation and the levels of pro-inflammatory cytokines, such as IL-6 and TNF-α. MSCs were identified in the tracheal aspirate of preterm newborns, showing phenotypical alterations compared to the MSCs from the control group of healthy babies [17,18]. The expression of proinflammatory cytokines in these cells was found to be higher compared to the control, strongly suggesting a role of MSCs in the pathology of BPD. Replacing these cells with exogenous MSCs could therefore represent a feasible treatment of BPD. This review focus on the mesenchymal and epithelial stem/progenitor cells.

## 2. Stem/Progenitor Cells in the Lung

In BPD, inflammation, oxidative stress, and mechanical ventilation can lead to abnormal lung growth and a reduced lung function. Studies have demonstrated that the number and function of stem and progenitor cells are altered in the lungs of infants with BPD [19]. Lung stem/progenitor cells belong to the endothelial, mesenchymal, and epithelial lineages [20]. The regeneration and repair of damaged lung tissue occurs by either the proliferation of already differentiated cells, or by the differentiation of stem/progenitor cells. Basal cells and variant club cells (VCCs) are present in the bronchi and in proximity of the neuroepithelial bodies, and they can give rise to club cells (CCs), ciliated cells, and goblet cells (GCs) [21]. In the alveoli, it is possible to identify bronchioalveolar stem cells (BASCs) that can differentiate into CCs, ciliated cells, GCs, and alveolar type 1 and 2 (AT1 and 2) cells, with AT2 cells having the ability to self-renew and give rise to the AT1 cells.

**Figure 1:**
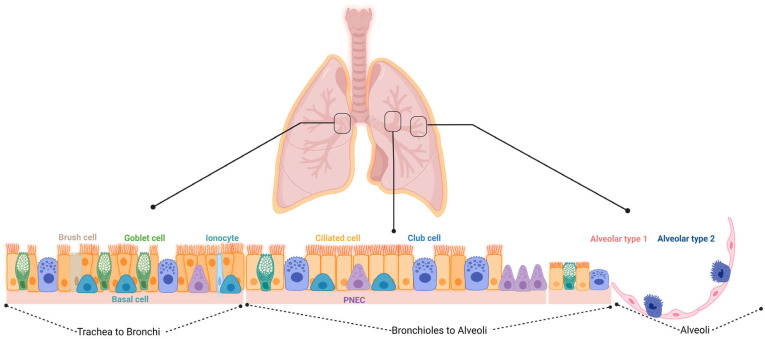
Schematic overview of the different cell types within the respiratory epithelium. Trachea to bronchi: the epithelial surfaces of the trachea and proximal airways are lined by a pseudostratified columnar epithelium consisting of basal, brush, ciliated, goblet, and club cells, along with the less frequent pulmonary neuroendocrine cells (PNECs) and ionocytes. Bronchioles to alveoli: small airways are lined by a simple columnar or cuboidal epithelium that consists of ciliated, club, and a few goblet cells. Alveoli: the alveoli are lined by either squamous alveolar type 1 (AT1) or cuboidal alveolar type 2 (AT2) cell.

The lung contains several different stem and progenitor cells, including basal cells (BCs), variant cells, GCs, BASCs, distal airway stem cells (DASCs), and AT2 cells (Figure 1 and Table 1 summarize the different localization of the cells and their specific markers). The activation of the stem/progenitor cells is associated with the level of lung injury. A severe injury of the alveolar epithelium leads to the activation of the non-alveolar epithelial type II cells to repair the damage; a mild injury induces the activation of the AT2 cells for the resolution of the damage. All these different types of cells could cover a potential therapeutic role in the treatment of BPD, including the MSCs and their extracellular vesicles (EVs) that have emerged as a promising therapeutic option as will be discussed further below.

### 2.1. Epithelial Progenitors

#### 2.1.1. Basal Cells (BCs)

BCs are the most primitive cell type in the airways and are capable of self-renewal and differentiation into other cell types, including ciliated, goblet, pulmonary neuroendocrine cells (PNECs), ionocytes, and club cells in steady-state and after acute lung injury to restore lung homeostasis [22,23,24,25]. They are present throughout the respiratory tree and account for about 30% of the total airway epithelial cells in the upper airways, while their number gradually decreases to 6% in the smaller airways, respectively [26,27]. Tumor protein (TP)63 and cytokeratin (KRT)5 are considered as the common markers to identify these BCs, along with podoplanin (PDPN) [28,29]. KRT8 is upregulated in the BCs just before they differentiate into luminal cells, generating an intermediate KRT8^+^ basal luminal precursor cell under homeostatic conditions [24,25,26]. Intermediate [29,30,31] luminal precursor cells can differentiate into secretory and ciliated cells and PNECs [25,32,33,34,35]. In addition to the KRT5^+^ progenitors, new KRT17^+^ basal progenitor cells were identified in the mouse airway epithelium using time-series single-cell transcriptomic analysis combined with in vivo lineage tracing [36]. It has been shown in murine models that basal cells are formed during prenatal development and contribute to the pool of luminal cells [37].

It seems that BCs play a role in regeneration and repair as well as they have been implicated in chronic lung disease. However, little is known about these cells in preterm neonates due to limitations in basal cell isolation techniques. A study has shown that these cells, isolated from the nasopharyngeal aspirates of preterm infants, could differentiate into the airway epithelium, thereby providing the possibility for future therapy in neonatal lung diseases like BPD [38].

#### 2.1.2. Secretory Cells (SCs): Variant Club Cells (VCCs), Club Cells, and Goblet Cells

CCs express secretory proteins (e.g., CC10) in addition to bronchiolar surfactants and proteases important for the regulation of epithelial integrity, immunity, and host-defense interactions [38,39,40]. A destruction and airway ciliated cell abnormality leading to a defect in mucociliary clearance has been found in premature infants developing BPD [40]. Moreover, ciliated cells of preterm infants showed a decline in ciliary beating, thus making the epithelium more vulnerable to external stimuli and injuries [38,41]. They are replenished by the basal cells, and their differentiation is maintained by Notch signaling [29]. It is possible to recognize two different types of CCs based on their sensitivity to naphthalene. The toxin induces the death of most of the CCs. However, a subpopulation is resistant to the injury, and is characterized by the lack of expression of the cytochrome P450 family member CYP2F2 [41]. These cells are the VCCs. They can reconstitute the injured epithelium through self-renewal and differentiation into the ciliated cells and CCs. VCCs can be considered as epithelial progenitors in small airways after injury.

GCs are secretory cells possessing numerous vesicle-bound mucin granules for mucus production, thus creating a mucous gel layer for the efficient mucociliary clearance and pathogen entrapment. Under healthy conditions, there is an equilibrium present between mucus production and clearance, however, in chronic airway diseases, hyperplasia and metaplasia of GCs can occur [41,42,43]. Moreover, they were found to metabolize xenobiotics via the production of P450 mono-oxygenase [44].

In infants with BPD, a decrease in the number of CCs as well as in the expression of the CC-derived protein secretoglobin (SCGB) 1A1 in the bronchiolar epithelium was reported [45,46,47]. This accelerated CC death might be a result of supplemental oxygen therapy in these infants. CCs are termed as the second stem cells in the airways since they serve as progenitors for ciliated and mucus-secreting cells.

Taken together, this could indicate a decrease in the levels of goblet cells in BPD because of CC hypoplasia, and hence this deficiency in the stem cell pool could lead to a lower host defense and interfere with the normal epithelial turnover and regeneration [48]. It has also been shown that GCs are plastic cells, and they may be able of self-renewal and trans-differentiation into a hybrid ciliated epithelial cell, thereby suggesting the presence of a transitional state [49].

#### 2.1.3. Pulmonary Neuroendocrine Cells (PNECs)

The first cells to be formed within the epithelium are the PNECs. They are present throughout the bronchial tree either as single cells in humans, or in clusters as neuroepithelial bodies in rodents, and their number increases from the bronchi to the terminal bronchioles [50,51,52]. A great number of PNECs is present in the fetal lung, suggesting that they play a role during fetal lung development [53]. Moreover, they are involved in the communication between the nervous and immune systems [53,54,55]. It has been shown that the number of PNECs as well as their peptide contents change in babies with BPD [56,57,58], which was also observed in a rodent model of hyperoxia. Additionally, increased levels of gastrin-releasing peptide (GRP)-, and calcitonin- and serotonin-immunoreactive PNECs have been reported in infants who died from BPD. In contrast, post mortem analysis of pre-term infants with respiratory distress syndrome revealed a decrease in the GRP^+^ PNECs that was assumingly caused by degranulation [56]. The overactivation and hyperplasia of PNECs have been reported in BPD, indicating a changed environment in BPD induced by chronic hyperoxia [56,59]. Hence, the dysfunction of pulmonary progenitor cells in BPD leads to an impairment in pulmonary growth and to the inability of the immature lung to repair itself [60].

#### 2.1.4. Alveolar Progenitor Cells

The distal alveolar region is lined with squamous type 1 and cuboidal 2 alveolar epithelial cells, also termed alveolar type 1 (AT1) and alveolar type 2 (AT2). AT2s are considered as alveolar progenitors with self-renewal abilities. Their turnover within the normal lung is slow, however, upon lung injury, they were shown to rapidly proliferate and differentiate into AT1 cells for maintaining the alveolar homeostasis [61,62,63,64,65,66]. The differentiation of AT2 into AT1 cells was observed in a study on neonatal rats under hyperoxia, and increased AT2 cell proliferation was observed in human severe type 2 BPD as well as in premature baboons following oxygen support and mechanical ventilation [67,68,69,70]. It is known that infants affected with BPD show a reduced formation of alveoli which continues to persist into adulthood [71,72]. Moreover, a decline in AT1 cells has been observed in infants with BPD, which is accompanied with alveolar simplification and the suspension of alveolarization [63,73].

Distal lung progenitor cells represent a subset of AT2 cells. They are responsible for the physiological turnover and for the repair through their ability to self-renew and differentiate into AT1 cells both in vivo and in vitro. In addition, they have regenerative potential: following pneumonectomy, an increase in AT2 cell numbers has been observed in mice, with a compensatory growth of the remaining lung parenchyma [74,75,76,77]. In the in vivo BPD model, hyperoxia exposure has been shown to deplete the AT2 cells [78]. This damage to the lung may contribute to an impaired or incomplete lung development and a resultant lifelong lung disease. The regenerative potential of AT2 cells therefore makes them interesting as a possible therapeutic intervention. In the in vivo model, primary murine AT2 cells were shown to prevent O_2_-induced functional and structural lung injury. Furthermore, it was demonstrated that induced human pluripotent stem cells also protected against lung injury in mice [79]. It has shown in past research how the intratracheal injection of progenitors can improve the structure and functions of the lung. Isolated AT2 cells were injected to prevent lung injury in an established oxygen-induced mouse model mimicking BPD. The results confirmed the safety of the administration and the efficacy of this procedure. However, the difficulty of isolating a sufficient number of these cells needs further work. Recently, different subpopulations of AT2 cells in terms of their activation status have been identified, thus increasing the complexity of a possible therapeutic approach with AT2 cells [80,81]

#### 2.1.5. Non-Alveolar Progenitors

The proximal airway progenitor cells include the already mentioned basal and secretory cells, while the distal progenitors comprise the VCCs and the non-alveolar epithelial type II derived progenitors, such as DASCs, BASCs, and lineage-negative epithelial progenitors (LNEPs).

#### 2.1.6. BASCs and DASCs

BASCs expressing the cell markers of both AT2 (surfactant protein C (SFTPC)) and secretory cells (secretoglobin 1A1 (SCGB1A1)) differentiate into AT1 and AT2 cells following bleomycin or influenza virus-induced injury, but not after hyperoxia-induced alveolar injury [41,82,83]. AT2 cells are capable of self-renewal and differentiation into AT1 (HOPX^+^) cells during homeostasis and upon injury [65,67]. In addition, AT2 cells differentiate into SFTPC^+^ and stem cell antigen (SCA)l^+^ alveolar progenitor cells, which are capable in differentiating into AT1 cells. In addition to the progenitor AT2 cells, another type of alveolar progenitor cells expressing alpha-6-beta4-integrin but not SFTPC and SCGB1A1 have been identified [84]. These cells can self-renew and differentiate into CCs and AT2 cells. Interestingly, it was found that a small population of AT1 cells expressing HOPX can give rise to AT2 cells following partial pneumonectomy [85]. A new population of stem cells distinct from AT2 and club progenitor cells has been identified, termed DASCs [86,87]. They express TP63/KRT5, can migrate, and give rise to SCGB1A1^+^/CYP2F2^+^ secretory club cells, which can self-renew and differentiate into ciliated cells and BCs (SCGB1A1^+^ and SFTPC^+^) upon lung injury.

#### 2.1.7. Lineage-Negative Epithelial Stem/Progenitors (LNEPs)

A lineage tracing study identified a very small subpopulation of quiescent club-like cells, characterized by a high expression of H2-K1 and a lack of TP63 that have the potential to differentiate into alveolar epithelial cells. These LNEPs are localized in the small distal airways and can contribute to the repair of the distal lung area [87].

**Table 1:** Summary of the specific markers that enable the precise identification of the epithelial stem/progenitor cell subpopulations present within the lung.

Cell Type	Location	Markers	Differentiation Potential	Refs.
Basal cells (BCs)	Epithelium in the trachea and bronchioles	TP63, KRT5, KRT14, NGFR, PDPN, KRT8, N2ICD, and c-Myb/TP73	Self-renewal; AT1, AT2 basal luminal precursor cell, neuroendocrine cells, ionocytes CCs, GCs, and ciliated cells	[23,87,88,89,90,91,92,93,94]
Variant club cells/ Clara cells	Bronchi and bronchioles (epithelium)	SCGB1A1 and CYP2F2^−^	Self-renewal: GCs and ciliated cells	[82,95,96]
Neuroendocrine cells (PNECs)	Epithelium, at branching points in the bronchi and bronchioles	GRP, ASCL1, CXCR4, NCAD and ROBO	Multiciliated cells and secretory cells	[49,55,97,98,99,100]
Broncho-alveolar stem cells (BASCs)	Epithelium and distal airway alveolar sacs	SCGB1A1 and SFTPC	Self-renewal: AT2 Ciliated cells: GCs and AT1	[101,102,103,104]
AT2	Distal airway alveolar sacs	SFTPC and LysM	Self-Renewal: AT1, SCA1^+^ mesenchymal progenitors, SFTPC^+^ AT1 progenitors,	[66,83,105,106,107,108]
Distal airway stem cells (DASCs), Alveolar progenitor	Distal airways, bronchioles, and alveolar regions	TP63 and KRT5	Self-Renewal: AT2	[84]
			Self-Renewal:	[86]
			AT2, SCGB1A1^+^, and CYP2F2^+^ CCs	[86,96]
AT1		HOPX,	AT2	[84]
Club cells, ciliated cells	Terminal bronchioles and less in the respiratory bronchioles, trachea, and bronchioles	SCGB1A1, and CYP2F2	BCs	[86]
			Ciliated cells	[96]
		TubIVa and FOXJ1	GCs	[109]
Integrin α6β4^+^ alveolar progenitor	Terminal bronchioles, broncho-alveolar junctions, and less in the respiratory bronchioles	SCGB1A1^−^, SPC^−^, ITGα6, and ITGβ4	Self-Renewal: AT1 Ciliated cells: AT2 and CCs	[96]

Abbreviations: ASCL-1, achaete-scute homolog 1; AT1/2, alveolar type 1/2 cells; BCs, basal cells; CCs, club cells; c-Myb, transcription factor; CYP2F2, cytochrome P450 family 2 subfamily f member 2; CXCR4, chemokine receptor; FOXJ1, transcription factor of fox family; GCs, goblet cells; GRP, bombesin or gene-related peptide; HOPX, homeodomain-only protein; ITG, integrin; KRT, cytokeratin; LysM, lysozyme M; N2ICD, Notch intracellular domain 2; NCAD, N-cadherin; NGFR, nerve growth factor receptor; PDPN, podoplanin; ROBO, roundabout receptor; SCA-1, stem cell antigen-1; SCGB1A1, secretoglobin 1A1; SFTPC, surfactant protein C; TP, tumor protein; and TubIVa, human tubulin beta class Iva.

## 3. Role of EVs—Secretome of MSCs

Extracellular vesicles (EVs), enveloped by the nuclear membrane, are 40–100 nm in diameter and are secreted by a variety of cells. Their components form the secretome of the respective cells of origin. EVs carry extracellular messages and mediate cell–cell communication [17,110,111].

The secretome of the MSCs consists of growth factors, cytokines, RNAs, and microRNAs, and differs depending on the tissue of MSC origin [112]. It can contribute to the beneficial effects of the EV on inflammation, repair, and organ development [113]. Their biological properties make MSCs very attractive for therapeutic use in the prevention and treatment of BPD [114]. This has already been assessed in several in vivo models with positive results on different diseases, such as in lung injury, cardiovascular disease, acute kidney injury, fetal hypoxic ischemic brain injury, wound healing, and hypoxic pulmonary hypertension [115,116,117,118,119]. Further studies have also shown that the use of MSC EVs alone does not lead to any disadvantages when compared to the complete MSCs [113]. EVs mimic their parental cells, MCSs, and they represent the major contributors of the paracrine effects of the MSCs. Moreover, EVs can be bioengineered to enhance their migratory properties [120].

Therefore, the use of MSC-derived EVs is a promising new therapeutic modality for BPD as it is a cell-free procedure, and thus can circumvent the concerns associated with viable MSC treatment [121]. However, before considering EVs as a therapeutic approach, additional preclinical studies are required to enlighten the effects on BPD and its underlying mechanisms, as well as to standardize the vesicle’s isolation and delivery.

## 4. Growth Factors and Inflammation in BPD

Risk factors for developing BPD include infections and injuries of the lung leading to inflammatory reactions. A major characteristic of BPD is the persistent inflammation contributing to impaired alveolarization and disturbing the cell–cell communication, as well as the correct response to the proliferation/differentiation signals. It is known that the dysregulation of several growths factors, like the vascular endothelial growth factor (VEGF), insulin-like growth factor 1 (IGF-1), PDGF, TGF-β1, and FGF10, plays an important role in the pathogenesis of BPD by affecting the inflammatory response. Several studies have shown an imbalance between the pro- and anti-inflammatory cytokines, which can predict the onset of BPD. For example, the concentration of pro-inflammatory cytokines has been shown to be increased in the amniotic fluid of preterm infants who develop BPD. Furthermore, cytokines are produced and secreted by several types of cells, are involved in cell–cell communications, and aid in modulating the answer to tissue damage.

## 5. Fibroblast Growth Factor 10 (FGF10)

FGF10 is a key growth factor for organ morphogenesis, including the lung [122]. During homeostasis in adult lungs, its expression is restricted to the mesenchymal niches and lipofibroblasts (LIFs), which are located close to the AT2 cells in the alveoli. LIFs, a fibroblast subpopulation, are emerging as a new possible source of FGF10 [123]. LIFs are characterized by the presence of lipid droplets, which are the main but not the only source of lipids to produce the surfactant lipoproteins in the AT2 cells [124]. Little is known about these cells, and even if they seem to be important in de novo alveologenesis during regeneration after lung injury [125], further studies are needed to define their role and relation with FGF10. In case of injury, induced by either bleomycin or influenza [126], FGF10 expression stimulates epithelial proliferation to promote the regeneration of the lung. FGF10 signaling is dysregulated in several lung diseases, including BPD [127].

In preterm infants with a severe or fatal course of BPD, a reduction in FGF10 levels has been observed. It has been published that in a mouse model of BPD (hyperoxia exposure) decreased FGF10 levels led to the premature death of the animals associated with profound alveolar defects and impaired AT2 cell differentiation, along with a decreased surfactant production [128]. Moreover, the administration of exogenous FGF10 to pups exposed to hyperoxia induced de novo alveologenesis. Furthermore, it has been published on how MSCs from the distal airways (LR-MSCs) of FGF10-pretreated rats can be easily isolated due to the mobilization effect of the FGF10 administration. LR-MSCs were injected into rat lungs with lipopolysaccharide (LPS)-induced acute injury, proving their protective role [129]. In these MSCs, FGF10 was additionally demonstrated to play a role in proliferation, mobilization, and organ-specific protective effects in acute lung injury [129]. Another in vivo study demonstrated that the administration of recombinant FGF10 induced de novo alveologenesis in a BPD mouse model [130].

Interestingly, it has been shown that FGF10 released from the mesenchyme correlates with an increased expression of VEGF in the epithelium [131].

## 6. Vascular Endothelial Growth Factor (VEGF)

VEGF regulates angiogenesis under both physiological and pathological conditions. The expression of VEGF is regulated by cytokines, such as IGF-1, but is also influenced by hyperoxia. This occurs via the mitogen-activated protein kinase (MAPK) and AKT signaling pathways, and thus impacts endothelial repair, regeneration, and vascularization. VEGF is secreted by MSCs and influences the differentiation of endothelial progenitor cells (EPCs). MSCs, can in turn differentiate into endothelial cells under VEGF treatment in vitro [132]. Interestingly, in vitro and in vivo hyperoxia experiments have shown that transplanted MSCs secrete VEGF, suggesting that this growth factor plays a role in protection against oxygen injury [133].

## 7. NF-κB

In the inflammation status observed in BPD and other lung injuries, the mesenchymal as well as the epithelial compartment is impacted by the cytokine storm, along with a dysregulation in several signaling pathways, like these of FGF10 and VEGF. In vivo models revealed that an increase in NF-κB is associated with lung injury [134]. Furthermore, it has been observed that hyperoxia, mechanical ventilation, oxidative stress, and endotoxins can lead to NF-κB activation [135,136,137]. One of several described anti-inflammatory modes of action of NF-κB is its ability to modulate macrophage polarization [138]. However, NF-κB can be either a pro-inflammatory or an anti-inflammatory factor based on the respective situation [139,140]. Many downstream target genes of NF-κB are important for innate and adaptive immune responses, and the activation of NF-κB has been described in various inflammatory diseases. In addition to these inflammatory processes, NF-κB regulates proliferation, differentiation, and angiogenesis [141,142]. As NF-κB mediates angiogenesis through the regulation of cytokines and growth factors, the inhibition of NF-κB has been described to block FGF-induced angiogenesis [142]. NF-κB regulates proliferation and apoptosis by controlling the transcription of several anti-apoptotic genes [143]. In an in vivo mouse model, NF-κB was shown to be constitutively active in newborn mice at the beginning of the alveolar stage, whereas it is only weakly expressed in the adult lungs [144]. The inhibition of the NF-κB pathway impairs alveolarization in neonatal mice through the inhibition of proliferation and the upregulation of apoptosis. Studies have indicated that the activation of NF-κB during the early stages of lung development can have a negative impact on development. For example, in lung explants at the late canalicular/early saccular stage, it has been shown that treatment with LPSs impedes lung branching [145]. This was thought to be due to NF-κB activation by LPSs in lung macrophages. Consequently, the depletion of macrophages or the inactivation of NF-κB could rescue lung branching [144], and NF-κB activation in macrophages resulted in an impaired branching [146]. NF-κB has been suspected to either act constitutively or to be induced otherwise. It has also been assumed that it acts differently at various stages of lung development. In preterm infants with BPD, an increased activity of NF-κB in tracheal lavage fluid could be detected [147]. Nevertheless, other studies have shown that a polymorphism in the *NF-ΚB IA* gene and a resulting increased activation of NF-κB reduces the risk of moderate-to-severe BPD [148,149]. Therefore, it has been suggested that NF-κB may play a protective role in late lung development [148].

MSCs and NF-κB are closely linked. MSCs isolated from preterm infants exhibit alterations in the PDGF-A, β-catenin, and TGF-β1 pathways, which are responsible for the MSCs in developing a myofibroblastic phenotype [150]. NF-κB can drive the proliferation of the MSCs, with the accumulation of the NF-κBp65 complex in the nucleus being the central regulatory mechanism. This is accompanied with a reduction in α-smooth muscle actin (α-SMA) expression, which is typical of the myofibroblastic phenotype [151]. However, we need to keep in mind that NF-κB pathway alterations, both activation and suppression, can lead to a disrupted lung development, emphasizing the intricate interplay between the NF-κB and other pathways in the cell. Due to the described impact on the MSCs and more generally on the inflammation, further analysis is needed to clarify the mechanisms underlying the interaction between NF-κB and the MSCs in order to balance the therapeutic approach to reduce the inflammatory process and its subsequent damages. Considering that NF-κB can exhibit both positive and negative impact on lung development, the time window along with the mechanisms of action on MSCs are crucial points [152].

## 8. Use of Stem Cells for the Treatment of BPD

Protocols for stem cell isolation and its subsequent in vivo administration are just barely developed far enough to be transferred from animal models to initial human clinical trials. A number of such studies have already addressed the safety and dose-responses with promising results, meaning that the next steps can focus more and more on therapeutic applications.

However, successful interventions still require a better understanding of these molecular processes, especially during differentiation of the cell types involved. This includes unique markers to improve cell-specific isolation, as well as a deeper knowledge of stem/progenitor cell physiology.

**Figure 2:**
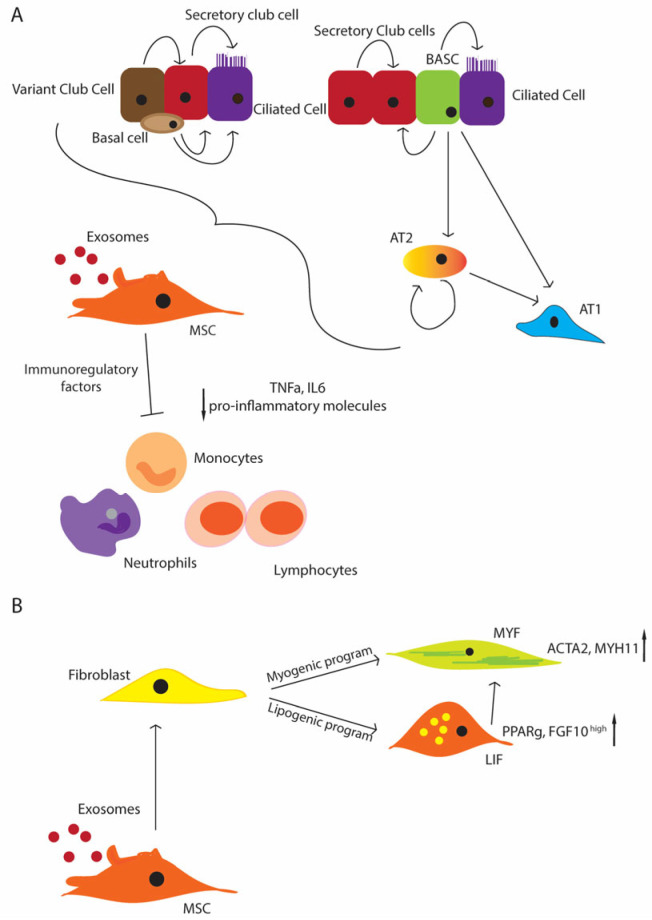
Schematic representation of the regenerative potential of the lung stem/progenitor cells (**A**) MSCs support the stem cell niche under physiological conditions and upon injury; basal cells differentiate in the secretory and ciliated cells. Variant cells can contribute to the secretory cell pool, and the secretory cells can come from the BASCs and secretory cells themselves. BASCs can differentiate in ciliated cells as well. AT2 cells can differentiate from the BASCs, and they represent the primary source of AT1 cells, and additionally they can maintain themselves; MSCs contribute to suppressing inflammation, inhibiting immune cells, such as monocytes, neutrophils, and lymphocytes (decreased level of pro-inflammatory molecules, TNF-α, IL-6 e.g.,) (**B**) MSCs give rise to the entire mesenchymal compartment, including lipofibroblasts (LIFs) and myofibroblasts (MYFs), with the activation of the lipogenic or myogenic program that can contribute to the progression or the resolution of injuries.

The mesenchymal compartment plays a fundamental role in supporting the proliferation and maintenance of the stem/progenitor niches in the lung (Figure 2A). Since MSCs give rise to different mesenchymal populations, like LIFs and myofibroblasts (MYFs), depending on the status of the tissues, the disturbance of their physiological function can cause an impaired lung development (Figure 2B). Thus, MSCs appear to be good candidates for stem cell therapy, and also because they are immune-privileged cells that do not induce host responses or cell rejection. Moreover, they can produce key molecules, such as FGF10 and PDGF [153,154], which influence the proliferation and differentiation of the stem/progenitor cells. As another advantage, they can be easily isolated from a wide range of tissues, like the bone marrow, peripheral blood, placenta, umbilical cord, and Wharton’s jelly [16,155]. The umbilical cord and Wharton’s jelly-isolated MSCs display more potent anti-inflammatory and immunomodulatory properties. Additional studies have focused more on the MSCs from the bone marrow and adipose tissues [15]. The safety profile of MSCs has been well established, and it is promising in that not only the MSC administration shows an improvement in the inflammation of the lung and its repair (e.g., alveolarization), but also of their EVs, suggesting a paracrine effect [112,113,114].

A recent study showed that a population of repair-supportive mesenchymal cells (RSMCs) rising from the GLI1^+^ cells is able to activate the club cell progenitors [156]. These RSMCs express high levels of FGF10 and enhanced WNT signaling. GLI1^+^ cells are usually located in the parabronchial, perivascular, and alveolar regions, and provide the FGF10-producing RSMCs in the lung. The comparison between the RSMCs and the resident MSCs (rMSCs) by scRNA-seq suggested the possibility that rMSCs could originate from the adjacent alveolar region surrounding the bronchi. Further investigations are needed to clarify whether this hypothesis is correct and transferable to human medicine [156].

To date, many animal studies have been published, highlighting a practical application of MSCs in the therapy of BPD, while only few clinical trials are being conducted. Nevertheless, from the preliminary results, one can be optimistic that within the next few years, we should be able to collect enough data to set up an effective therapeutic approach for BPD. In the next sections, we will focus on the most promising studies.

## 9. Animal Studies

Numerous studies in animal models provide evidence of the beneficial use of stem cell therapy in BPD, with MSCs being the most extensively used cell type [157,158,159]. The benefits of MSC therapy include reduced pulmonary fibrosis and edema, restoration of lung function, and attenuation of lung inflammation [160,161,162,163]. Considering the perturbation of stem cells in BPD, the repopulation, repair, and regeneration of the lung with healthy stem/progenitor cells would be an ideal therapeutic approach. Several studies have shown that intratracheal, intravenous, and intraperitoneal administrations of bone-marrow MSCs (BMSCs) have prevented alveolar arrest and attenuated lung inflammation in a neonatal lung injury model [164]. Additionally, this treatment has stimulated other stem/progenitor cells, termed BASCs, to aid in lung repair [164,165]. A recent study on BMSC administration in a mouse model of BPD demonstrated the ability of the cells to migrate from the site of the i.p. injection to the injured lung, resulting in an improvement in the pulmonary architecture, a reduction in pulmonary fibrosis, and an increase in the survival rate [166]. Mice were exposed to hyperoxia for a period of two weeks and were then treated with an intravenous dose of MSCs-conditioned media (MSC-CM). Subsequently, a reversal of hyperoxia-induced parenchymal fibrosis, partially reversed alveolar injury, and fully reversed pulmonary hypertension was observed, thus showing that MSCs-secreted factors can support the reversal of symptoms associated with hyperoxia-induced BPD [167]. In accordance with these findings, a similar study used intratracheal injections of umbilical cord-derived perivascular cells and cord blood-derived-MSCs in rat pups exposed to hyperoxia [155]. The administration of both cell types resulted in an improved alveolar architecture, thus partially restoring the lung function. Comparable results were obtained when using MSC- or PC-conditioned medium, suggesting that the therapeutic outcome observed was due to a paracrine effect [155].

Another cell type to achieve lung repair could be human amnion epithelial cells (hAECs). Administration of these cells in an animal model of LPS-induced injury in fetal sheep ameliorated pulmonary inflammation [168].

## 10. Clinical Trials

As previously described, in vivo hyperoxia studies in rodents demonstrated that the administration of MSCs resulted in an improvement in the lung structure of hyperoxia-damaged lungs [81]. This effect was even more pronounced when MSC-conditioned medium was administered [165,167], suggesting that MSCs or their secreted components ameliorate the imbalance of the inflammatory response and cytokines in oxygen-damaged lungs. These promising results led to the use of MSCs in clinical trials.

It has been described that in the tracheal aspirates of preterm infants who later developed severe BPD, TGF-β1 activity is significantly increased [18]. The MSCs of preterm infants with BPD express TGF-β1 and possess stronger β-catenin signaling compared to MSCs from infants who do not develop BPD [169]. Studies have shown that TGF-β1 and the nuclear translocation of β-catenin control the MYF differentiation [154,169]. In in vivo models, hyperoxia has been shown to damage the function of lung-resident stem cells with mesenchymal, endothelial, and epithelial differentiation potential [81]. These studies indicate that damage to MSCs in the lung may promote BPD [85].

In recent years, stem cells have been increasingly used in clinical trials not only relating to BPD. Currently, the results of three completed phase I clinical trials in preterm infants with BPD have been published, and a number of clinical trials (phase I and II) are either open for recruiting, ongoing, or have recently been completed. They are focusing on the benefit and safety of stem cell transplantation in BPD patients (NCT02443961; NCT03378063; NCT04003857; NCT03873506; NCT03631420; NCT03645525; and NCT03774537, respectively).

A phase I dose-escalation study examined the safety and feasibility of the intratracheal administration of MSCs in nine preterm infants with a mean birth weight of 793 ± 127 g and mechanical ventilation support being at high risk of developing BPD [170]. Three patients were given a low dose (1 × 10^7^ cells/kg), and six patients were given a high dose (2 × 10^7^ cells/kg), respectively. In tracheal aspirates at day 7 after MSC administration, a decrease in the pro-inflammatory cytokines TNF-α, IL6, IL8, and matrix metalloproteinase 9 levels was found, going in line with a lower severity of respiratory disease [170].

In a second phase I study, MSCs from the umbilical cord were administered to twelve preterm infants with a birth weight of <1000 g and a gestational age <28 weeks, respectively. While intratracheal treatment was well tolerated, ten infants developed severe BPD, and two encountered a mild BPD, respectively. In addition, three cases of sepsis were recorded [158]. In this study, premature infants were less mature, and had more severe respiratory disease compared to the first study, which may explain the poorer outcome.

In the third phase I study, the safety of late allogeneic amniotic cell transplantation was investigated in infants with severe BPD who either required invasive ventilation or non-invasive respiratory support with oxygen fractions of 0.3–0.5 [171]. In total, six premature infants were given a dose of 1 × 10^6^ cells/kg, and after a slight adaptation of the procedure after the first patient, five infants tolerated the infusion without experiencing side effects. The treatment did not lower the need for respiratory support in any of the patients within the first week. However, FiO_2_ requirements decreased in three infants [171]. Although no treatment-associated adverse events were observed up to 24 months of age, the significance remained limited due to the severe BPD of the infants and the persistent problems of somatic growth, and cardiorespiratory and psychomotor function [172].

The subsequent phase I dose-escalation study has already begun, in which the administered cell number was increased up to 10 × 10^6^ cells/kg per infusion and up to 3 × 10^7^ cells/kg in total, respectively [173].

These first three phase I trials have primarily focused on the safety aspects of MSC administration and did not consider the overall benefit since the application and dosage or extraction of the MSCs must first be clarified for a successful MSC therapy. It has been shown that MSC treatment has no side effects in preterm infants and does not contribute to deterioration. However, the long-term effects of MSCs administration for more than two years has not yet been investigated.

Taken together, these clinical trials indicate that the safe administration of MSCs and any benefit from this treatment are likely to depend on several factors. The dose and timing of MSC administration and repeated treatments appear to be important in this regard, each alone and in combination. However, these factors have not yet been studied in detail.

## 11. Summary and Conclusions

The technical advance in the cell analysis leads to a better understanding of cell diversity, thereby improving the knowledge on the stem/progenitor cells present in the different regions of the lung. Alveolar progenitors and epithelial progenitors from the amniotic fluid are not yet well characterized to be translated in clinical research. Recent findings highlighted the MSCs as a possible therapeutic option to improve the damage occurred in the lung in case of injury, like virus-induced, hyperoxia-induced, or mechanical-induced injuries. MSCs seem to act via paracrine mechanisms, supporting the central role of their EVs. EVs contain a variety of mRNA, lipid, and proteins, and its content could be the key to the beneficial effects observed in the experiments. The administration of both the MSCs and their eVs showed positive outcomes, with an improved respiratory function and lung structure. However, there is still not enough knowledge in terms of the cell preparation, the number of cells, the time points, the type of administration, and the long-term studies on their safety. MSCs from the umbilical cord represent the best therapeutic option to be translated for clinical usage. One of the major obstacles is related to the expansion of the MSCs in vitro, as it is barely possible to keep track of the changes in the cells leading to an altered behavior. For this reason, the use of EVs or MSC-conditioned media could be an alternative. So far, the application of both is limited to animals.

Taken together, stem/progenitor cell-based therapies are very promising to counteract the damage in the injured lung. MSCs are the most extensively studied type of cells for this therapeutic application, and the preliminary studies are very promising. However, first, it is necessary to fill the gap of knowledge in the understanding of the MSC’s behavior, as well as to optimize/standardize the isolation protocol and their administration for future clinical trials.

## Data Availability

Not applicable.
